# Distraction in diagnostic radiology: How is search through volumetric medical images affected by interruptions?

**DOI:** 10.1186/s41235-017-0050-y

**Published:** 2017-02-20

**Authors:** Lauren H. Williams, Trafton Drew

**Affiliations:** Department of Psychology, University of Utah, Salt Lake City, UT USA

**Keywords:** Radiology, Interruptions, Visual search, Volumetric medical images, CT scan, Eye-tracking, Healthcare

## Abstract

Observational studies have shown that interruptions are a frequent occurrence in diagnostic radiology. The present study used an experimental design in order to quantify the cost of these interruptions during search through volumetric medical images. Participants searched through chest CT scans for nodules that are indicative of lung cancer. In half of the cases, search was interrupted by a series of true or false math equations. The primary cost of these interruptions was an increase in search time with no corresponding increase in accuracy or lung coverage. This time cost was not modulated by the difficulty of the interruption task or an individual’s working memory capacity. Eye-tracking suggests that this time cost was driven by impaired memory for which regions of the lung were searched prior to the interruption. Potential interventions will be discussed in the context of these results.

## Significance

Radiologists are frequently interrupted during the interpretation of medical images. The current research provides the first attempt to quantify the effect of these interruptions using an experimental design. In our study, we found that interruptions lead to a significant increase in task completion time. Through the use of eye-tracking, we were able to determine that this inefficiency is driven by impaired memory for previously searched areas of the image. In natural settings, these results translate to longer patient turnaround times and increase the cost of providing and receiving healthcare. By establishing a causal link between interruptions and productivity loss, we aim to encourage healthcare providers to reduce unnecessary interruptions in radiology reading rooms. In addition, our eye-tracking results hint at potential interventions, such as eye-tracking feedback, that may help lower the cost of unavoidable interruptions.

## Background

Interruptions have been identified as a prevalent and potentially harmful occurrence in radiology reading rooms. A recent workflow analysis found that radiologists are interrupted once every 12.1 min on average during regular business hours (Ratwani, Wang, Fong, & Cooper, 2016). These interruptions are primarily in the form of medical questions during in-person or phone-call interactions. During after-hours radiology, interruptions may be even more common. At many academic institutions, after-hours phone calls are handled by a single radiology resident (Balint et al., 2014). A recent study found that on-call radiologists receive an average of 72 phone calls during a typical 12-h overnight shift (Yu, Kansagra, & Mongan, 2014). This rate of interruption equates to a 59% chance of being interrupted by a phone call for every 10 min spent reading a computed tomography (CT) scan. In a separate analysis of after-hours reading environments, increases in phone-call volume were associated with an increase in the number of errors made by radiology residents (Balint et al., 2014). Similarly, interruptions have been linked to medical errors in other tasks, such as dispensing medication (Westbrook, Woods, Rob, Dunsmuir, & Day, 2010).

The significance of a potential link between interruptions and medical errors is difficult to overstate. In 2000, the Institute of Medicine’s *To Err is Human* report implicated medical errors in almost 100,000 deaths and over 1 million injuries in America each year (Kohn, Corrigan, & Donaldson, 2000). Although there is no official count of these casualties, more recent estimates have placed medical errors as the third leading cause of death in America (Makary & Daniel, 2016). In addition to injury and loss of life, medical errors are a substantial financial burden to society. Diagnostic errors are the leading cause of successful medical malpractice litigation and result in the highest payout per case (Tehrani et al., 2013; Whang, Baker, Patel, Luk, & Castro, 2013). Compared to doctors from other specialties, radiologists are disproportionately named as defendants in these claims (Physician Insurers Association of America, 2004; Whang et al., 2013).

Interruptions have also been linked to decreased productivity in the workplace. Over the ten-year period from 1999 to 2010, the institutional workload for radiologists increased tenfold (McDonald et al., 2015). After adjusting for increases in staff over this period, the number of images that need to be interpreted increased from 2.9 to 10.1 per minute. Faced with this increasing workload, radiologists are under substantial pressure to maintain productivity. In the workflow analysis by Ratwani et al. (2016), the mean time spent handling each interruption was 2.4 min. In 10.6% of these interruptions, the secondary task was completely unrelated to medicine. It is likely that all interruptions have negative consequences on productivity, but these unrelated interruptions might be particularly problematic. Task-switching research has consistently demonstrated that interleaving multiple tasks takes more time than completing each of the tasks separately (for a review, see Monsell, 2003). This time cost is typically on the order of milliseconds in laboratory tasks, but these results have been replicated on a larger scale in a number of applied settings. For example, task-completion time doubled when telecommunications workers were interrupted by a secondary task (Eyrolle & Cellier, 2000).

In recent years, advancements in medical imaging technology have dramatically increased the size and complexity of the radiologist’s workload. Two-dimensional (2D) film images, such as chest radiographs, have largely been replaced by volumetric images such as CT or positron emission tomography (PET) scans. These images consist of hundreds, and sometimes even thousands, of images stacked together to form three-dimensional (3D) representations of the body (Andriole et al., 2011). By most accounts, these imaging techniques have led to positive patient outcomes (Mathieson, Mayo, Staples, & Müller, 1989; National Lung Screening Trial Research Team, 2011). However, the effects of interrupting radiologists during these large, complex images are unknown. To the extent that medical images are analogous to laboratory visual search paradigms, we can gain insight from the literature on interrupted visual search. Spatial memory, which we define as memory for locations in a visual scene, is thought to play an important role in the successful resumption of an interrupted visual search task. Primary support for this idea comes from the rapid resumption literature, which demonstrates that interrupted search is resumed more quickly than a new search can be initiated (Lieras, Rensink, & Enns, 2005). These results suggest that memory for the scene is retained throughout the interruption and is therefore able to facilitate task resumption. However, the interruptions used in these paradigms are typically brief, unfilled time delays. More complex interruptions, such as secondary search tasks, have been shown to disrupt memory for visual search arrays after only a few seconds, and this memory seems to be completely eradicated by interruptions with longer durations (Shen & Jiang, 2006). These results are consistent with known constraints on suspected mechanisms of memory in visual search, such as inhibition of return, which is limited in both capacity and duration (for a review, see Wang & Klein, 2010). Although spatial memory in visual search seems to be relatively fragile, the ability to remember where you were in a task has also proven to be a key component in resuming interrupted computer tasks (Ratwani, Andrews, McCurry, Trafton, & Peterson, 2007; Ratwani & Trafton, 2008). In large volumetric images, it may be difficult to maintain these important spatial representations of the task during an interruption. This impaired memory could have a negative impact on task completion time and error rate by causing regions of the image to be unnecessarily revisited or completely overlooked after an interruption.

In the human-computer interaction literature, Altmann and Trafton’s (2002) Memory for Goals model has been a useful framework for understanding and predicting the effects of interruption. According to this model, the success of task resumption is dependent on the relative activation level of goal-relevant information in memory. When a primary task is interrupted, relevant information about the task must be temporarily stored in memory. In order to resume the task, this goal-relevant information must be retrieved from memory. This model predicts that goals with greater activation will be retrieved more quickly and have a smaller time cost. The strength of goal activation is constrained by three factors: interference (e.g. strength of irrelevant goals), strengthening (e.g. goal rehearsal), and priming (e.g. cues in the environment). This model makes many predictions about interruptions that have been successfully tested in the literature (Altmann & Trafton, 2004; Chung & Byrne, 2008; Hodgetts & Jones, 2006; Monk, Boehm-Davis, Mason, & Trafton, 2004; Monk, Trafton, & Boehm-Davis, 2008; Trafton, Altmann, & Brock, 2005; Trafton, Altmann, Brock, & Mintz, 2003). Although the majority of these findings have been in human–computer interaction tasks, this model may also be an effective framework for understanding the effects of interruptions in visual search tasks.

Radiologists work in an increasingly complex and highly disruptive environment. Despite indications that interruptions might be both harmful and frequent in this environment, the effects of these interruptions have yet to be examined using an experimental design. The purpose of the current research is to quantify the cost of interruptions in diagnostic radiology in terms of error rate and search time. In addition to these behavioral measures, eye-tracking will be used to gain a qualitative understanding of how interruptions affect search through volumetric images. We anticipate that interruptions will lead to an increase in errors and search time. Based on the existing literature, these effects are expected to be driven by impaired memory for which regions of the image were searched prior to the interruption.

## Experiment 1

### Materials and methods

#### Participants

Twenty-nine students from the University of Utah participated in the study for course credit or $10 an hour. The experimental design was approved by the Institutional Review Board and all participants provided informed consent. Data from three participants were discarded prior to analysis: two for uncorrected vision impairments and one for completing the majority of trials too quickly to reach the experimental condition. Twenty-six participants were included in the data analysis (16 women, mean age = 21.6 years, age range = 18–42 years). Due to difficulty with calibration, eye-tracking data are missing for one participant. Participants were not medically trained and had no experience interpreting medical images prior to the study.

#### Primary task

Chest CT scans are volumetric representations of the lung that consist of stacked axial images. During lung cancer screening, radiologists search for small nodules that “pop in and out of view” as the reader scrolls through the depth of the image. In the current study, participants searched through 21 (1 practice, 20 experimental) chest CT scans for nodules. The CT scans had a 1024 × 1024 resolution and were centered on a 1920 × 1080 monitor. The images subtended approximately 25° of visual angle. Each CT scan consisted of 51 lung slices and 2 to 11 artificially embedded nodules that had a diameter in the range of 18–26 pixels. The up and down arrow keys were used to freely scroll through the depth of the lung. Participants were instructed to search the lung thoroughly and mark detected nodules using the computer mouse. There was an unlimited amount of time to view each CT scan and search was self-terminated by clicking on a box located on the side of the screen. Each participant underwent an instructional period and a practice trial before proceeding to the experiment. Task stimuli were presented using the Psychophysics toolbox in Matlab (Brainard, 1997).

#### Interruption task

**Fig. 1:**
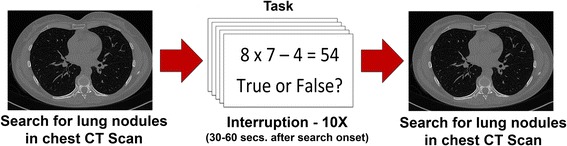
*Layout* of an interruption trial

Half of the trials were interrupted by a series of ten true or false math equations (see Fig. 1). Each CT scan was assigned a specific interruption time in the range of 30–60 s following search onset. The math equations were randomly generated with numbers between 1 and 10 using the format A * B – C = D. Each set of problems was solved correctly 50% of the time. Participants responded using the right arrow key for correctly solved equations and the left arrow key for incorrectly solved equations. The screen flashed red for 100 ms following an incorrect response.

Participants were instructed to be as accurate as possible and to treat both tasks with equal importance. CT scans were divided into two groups such that an equal number of participants were interrupted on each group of images. This design ensures that any observed effect is due to the interruptions rather than any differences in difficulty across CT scans. The images were presented in a random order and participants did not know if or when a case would be interrupted at the start of each trial.

#### Eye-tracking

Eye-movements were recorded using the Eyelink 1000 Plus (SR Research, Ontario, Canada). Participants were positioned in a chinrest approximately 64 cm away from the computer. A nine-point calibration procedure was performed every five to seven trials. Eye position was sampled at 500 Hz. In order to obtain x, y, and z coordinates for the volumetric images, the position in depth was co-registered at each time point using Eyelink messages.

### Behavioral results

#### Math performance

On average, participants spent 38.2 s completing the math problems. The problems were solved correctly 90% of the time.

#### Search time

**Fig. 2:**
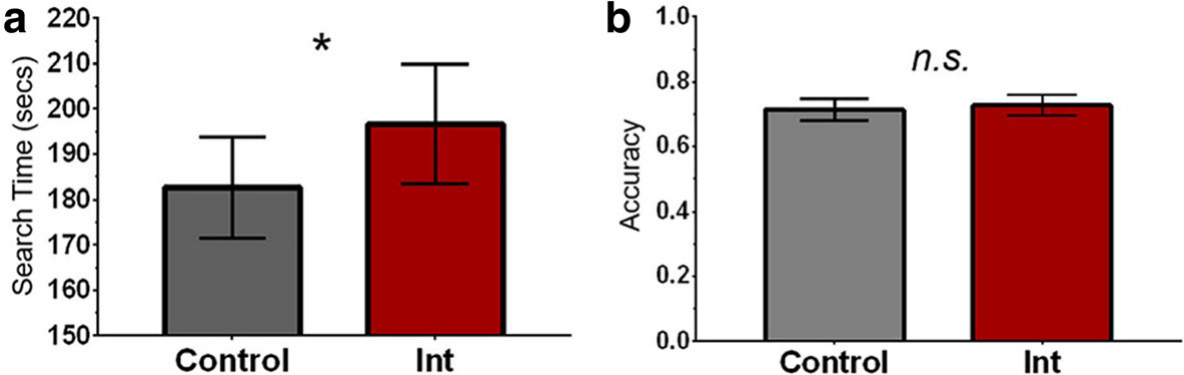
**a** Search time results for Experiment 1 by condition. **b** Nodule detection rates for Experiment 1 by condition. The *star* denotes significance (*p* < 0.05) and the *error bars* are the standard error of the mean

After accounting for the amount of time spent on math problems, participants spent significantly more time searching interrupted cases (M = 196.66 s, SD = 67.9 s) than control cases (M = 182.66 s, SD = 56.81 s), t(25) = 2.62, *p* = 0.015, Cohen’s *d* = 0.22 (see Fig. 2a). The average time cost was 14 s (median: 10.68 s, range: –37 to 88 s), which is an 8% increase in search time for interrupted cases.

#### Accuracy

Overall, 70% of the nodules were detected (see Fig. 2b). There were no significant differences in the number of missed nodules per lung between interrupted (M = 1.82, SD = 0.78) and control cases (M = 1.89, SD = 0.78), t(25) = 0.76, *p* = 0.45, Cohen’s *d* = 0.09. False alarms were infrequent and the number of false alarms per lung did not differ between interruption (M = 0.05, SD = 0.09) and control cases (M = 0.08, SD = 0.18), t(25) = 1.27, *p* = 0.21.

### Eye-tracking results

#### Useful field of view

In order to calculate lung coverage and refixation rate, we need to estimate the useful field of view (UFOV) in a volumetric image. The UFOV is the area around a foveated point that can be attended without moving the eyes (Ball, Beard, Roenker, Miller, & Griggs, 1988). In the literature, a 5° diameter estimate has been used to study search through 2D medical images (Kundel, Nodine, & Krupinski, 1989; Nodine, Mello-Thoms, Kundel, & Weinstein, 2002). However, UFOV is known to decrease with the complexity of the stimuli and it is unknown if the added depth dimension in CT scans changes this estimate (Young & Hulleman, 2013). Furthermore, novices may not be able to extract as much information in a single fixation as expert radiologists (Krupinski, 2012; Kundel & La Follette Jr, 1972; Manning, Ethell, Donovan, & Crawford, 2006). To account for these factors in our study, we used a smaller (2.5°) estimate in our calculations, which increases the precision of the measure and is closer to most estimates of foveal vision (Wandell, 1995).

#### Coverage

**Fig. 3:**
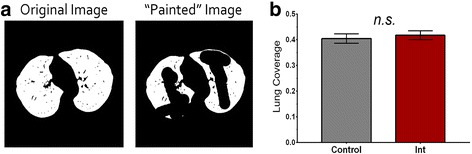
**a** *Illustration* of lung coverage analysis. Each *image* was converted to a *black and white image*, where *white pixels* are the lung tissue and *black pixels* are the non-lung regions. *Black pixels* were “painted” on the original image for every set of coordinates searched. Final coverage was calculated as follows: 1 – (number of white pixels in painted image/number of white pixels in original image). **b** Percentage of lung covered in each condition. *Error bars* represent the standard error of the mean

Lung coverage was calculated using the x, y, and z coordinates for each sampling point. Image processing applications in Matlab were used to create black and white versions of each lung slice. White pixels represented lung tissue and black pixels represented areas that were not lung tissue. For each lung slice, a new image was generated with black circles centered at each set of visited coordinates (see Fig. 3a). Each circle subtended 2.5° of visual angle. Lung coverage was calculated as 1 minus the percentage of white pixels remaining in the new image out of the number of white pixels in the original image. There were no significant differences in lung coverage between interruption (42%) and control (40%) trials, t(24) = 1.83, *p* = 0.08, Cohen’s *d* = 0.13 (see Fig. 3b).

#### Search resumption

The accuracy of search resumption was calculated by dividing the lung into quadrants and comparing the locations of the last pre-interruption fixation and the first post-interruption fixation. If memory is retained following the interruption, search should resume somewhere in the vicinity of the most recently searched region of the image. However, search was resumed in the correct quadrant only 23.1% of the time, which is statistically equivalent to chance, t(24) = 0.63, *p* = 0.534. Furthermore, the rate of inaccurate search resumption (M = 76.9%) is significantly greater than the overall rate of quadrant changes between consecutive fixations (M = 27.6%), t(24) = 11.43, *p* < 0.001. In other words, the two fixations surrounding the interruptions are in different quadrants of the lung far more often than consecutive fixations during typical search. This suggests that the high rate of quadrant changes between pre and post interruption does not reflect an overall tendency to frequently switch quadrants of the lung during search. Instead, these results suggest that interruptions impair memory for which region of the lung was searched immediately prior to the interruption.

#### Refixation rate

**Fig. 4:**
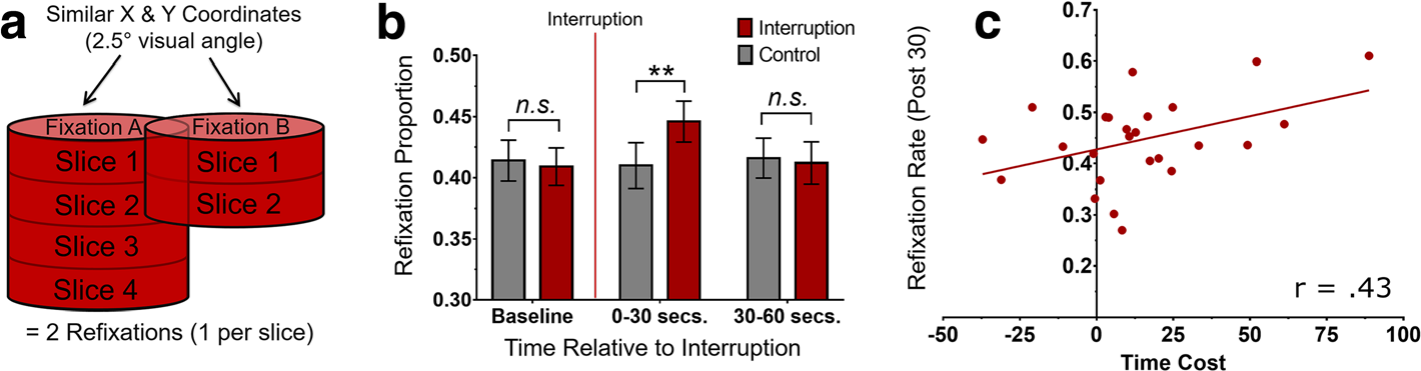
**a** *Illustration* of refixation analysis in volumetric space. **b** The percentage of refixations out of total fixations in each condition. Each trial was separated into 30 s epochs relative to the time the interruption occurred. *Double stars* indicate significance (*p* < 0.01) for the within-subject comparison between interruption trials and the equivalent time period in control trials. *Error bars* denote the standard error of the mean. **c** Correlation between refixations in the 30 seconds following interruption and the overall time cost

Traditional eye-movement classifications, such as fixations and saccades, do not have a clear definition in volumetric space. For example, a reader may scroll through several layers of depth while maintaining their gaze at a fixed x and y position. Although eye-tracking software would classify this as a fixation, it is not a fixation in the traditional sense. For current purposes, each eye-tracker defined fixation was treated as a cylinder that permeated each layer of the lung that was visited during that time period (see Fig. 4a). The base of the cylinder was a circle that subtended 2.5° of visual angle. Each time a cylinder overlapped with another cylinder, it was classified as a refixation. In order to account for the time differences between trials, we calculated refixation rate instead of the absolute number of refixations. Refixation rate was defined as the proportion of total fixations that fell within 2.5° of a previous fixation during a given time period. In other words, refixation rate is a measure of how frequently previously viewed spatial locations are searched relative to novel spatial locations.

Each individual CT scan was associated with a unique interruption time, which allowed us to compare equivalent time periods across interruption and control trials. For example, if one group of participants was interrupted during Scan X at 40 s and the other group was not interrupted during Scan X, the refixation rate for Scan X would be calculated relative to the 40-s time point for each group. The critical comparisons were between the refixation rates for the interruption trials and the control trials during the same time periods for a given scan. During the 30 s prior to the interruption time for each CT scan, there were no significant differences in refixation rate between interruption (M = 0.41, SD = 0.08) and control (M = 0.41, SD = 0.08) trials, t(24) = 0.52, *p* = 0.61. However, we found a significant difference in refixation rate between interruption (M = 0.45, SD = 0.08) and control trials (M = 0.41, SD = 0.09) in the 30 s immediately following the interruption time, t(24) = 3.151, *p* = 0.004, Cohen’s *d* = 0.41 (see Fig. 4b). In the 30–60-s post-interruption period, the difference in refixation rate between interruption (M = 0.41, SD = 0.08) and control trials (M = 0.42, SD = 0.08) returned to baseline, t(24) = 0.36, *p* = 0.72.

For interruption trials, the refixation rate during the 0–30-s time period significantly correlates with each individual’s average time cost (search time for interruption trials – search time for control trials), r(23) = 0.43, *p* = 0.03. This correlation is not significant at baseline or in the 30–60-s time window. Furthermore, a median split of the data reveals a significantly higher refixation rate in the post-interruption period for the participants with the greatest time cost (M = 0.48, SD = 0.08) than the participants with the smallest time cost (M = 0.41, SD = 0.08), t(23) = 2.34, *p* = 0.029.

## Experiment 2

In Experiment 1, interruptions led to an increase in task completion time. Although one would expect longer search times to lead to increased lung coverage and fewer missed nodules, there were no differences across the two conditions. The eye-tracking measures revealed that this inefficiency of search seems to be driven by impaired spatial memory during the time period immediately following the interruption. In Experiment 2, we sought to determine which features of interruptions might modulate the associated time cost. The Memory for Goals model (Altmann & Trafton, 2002) suggests that the difficulty of the interruption task will influence the magnitude of the interruption cost. According to this account, difficult interruptions impair the ability to maintain goal-relevant information during an interruption to a greater extent than easy interruptions. Therefore, we should observe a greater time cost if we increase the difficulty of the interruption task. However, Cades, Davis, Trafton, and Monk (2007) emphasize that it is the ability to rehearse goal-relevant information, rather than subjective task difficulty, which predicts the disruptiveness of an interruption. Based on this more nuanced interpretation of the Memory for Goals model, we might not observe any additional time in more difficult interruption tasks. In Experiment 1, the accuracy of search resumption was statistically equivalent to chance. This suggests that the ability to rehearse relevant spatial information was impaired by the interruption. Once the opportunity to rehearse has been eliminated, more difficult interruptions might not place any additional demands on the participant. However, if there is an effect of task difficulty beyond the ability to rehearse, we should observe an increase in time cost for difficult interruptions.

In addition to the difficulty manipulation, we administered a visuospatial working memory task to determine if individual differences in working memory capacity explain the variation in interruption cost. Working memory, perhaps more than any other cognitive measure, has been linked to meaningful outcomes, such as reading comprehension (Daneman & Carpenter, 1980), academic performance (Colom, Escorial, Shih, & Privado, 2007), and fluid intelligence (Kane, Hambrick, & Conway, 2005; Unsworth, Fukuda, Awh, & Vogel, 2014). Most notably, working memory capacity explains individual variation in multitasking ability (Redick, 2016). Individuals with high working-memory capacity might have the cognitive resources to better maintain task-relevant information in memory throughout an interruption. According to the Memory for Goals framework (Altmann & Trafton, 2002), this ability would allow these individuals to resume the task more quickly. Therefore, we expect to observe a negative correlation between working memory capacity and time cost.

### Methods

#### Participants

Thirty-two students from the University of Utah participated in the study for course credit or $10 an hour. Each participant provided informed consent prior to study participation. Data from five participants were discarded prior to the analysis: one for a program malfunction and four because they did not complete the study in the allotted time. Twenty-seven participants were included in the data analysis (18 women, mean age = 25.3 years, age range = 18–39 years). As in Experiment 1, participants had no medical experience prior to the study.

#### Working memory task

At the beginning of the experiment, each participant completed a change detection working memory task (Luck & Vogel, 1997). While fixating on a central cross, colored squares (set sizes 2, 4, and 8) flashed on the screen for 100 ms. During the test phase, one of the squares reappeared in its original location but changed color in 50% of the trials. The participants’ task was to indicate whether the square had changed color using the “F” (same) or “J” (different) key. Working memory capacity was calculated using Cowan’s K formula, K = (hit rate + false alarm rate) * N (Cowan, 2001).

#### Primary task

Participants searched through 22 (1 practice, 21 experimental) chest CT scans for nodules using the same procedure described for Experiment 1. No eye-tracking data were recorded in Experiment 2.

#### Interruption task

Experiment 2 had three conditions: easy interruptions, hard interruptions, and no interruptions. Two of these conditions (no interruptions and easy interruptions) were identical to the tasks described in Experiment 1. The hard interruptions were in the same format, A * B – C = D, but the A and C positions were replaced with numbers between 10 and 19. There were seven trials for each condition and each individual CT scan was assigned to each condition an equal amount of times across participants.

### Results

#### Math performance

On average, participants spent 40.59 s completing the easy math problems and 66.45 s on the hard math problems, t(26) = 4.71, *p* < 0.001, Cohen’s *d* = 1.03. There were significantly more errors for the hard (M = 2.49, SD = 1.92) math problems than the easy math problems (M = 1.6, SD = 1.89), t(26) = 4.92, *p* < 0.001, Cohen’s *d* = 0.47.

#### Search time

**Fig. 5:**
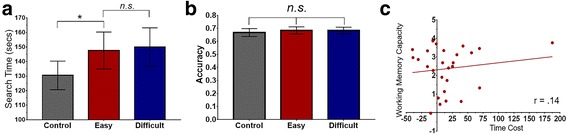
**a** Difference in search time across conditions. Stars indicate significance (*p* < .05). Errors bars are the standard error of the mean. **b** The percentage of nodules found in each condition. **c** Correlation between working memory capacity and time cost

The time cost observed in Experiment 1 was replicated in Experiment 2. After subtracting the time spent on the math problems, interruption trials (M = 148.2, SD = 63.29) were searched significantly longer than control trials (M = 130.48, SD = 51.08), t(26) = 2.08, *p* = 0.048, Cohen’s *d* = 0.31 (see Fig. 5a). The average time cost was 18 s (median = 11 s, range = –40 to 187.5 s), which is a 13% increase in search time. Difficult interruption trials (M = 149.9, SD = 68.85) were not associated with any additional time cost relative to the easy interruption trials (M = 147.58, SD = 66.16), t(26) = 0.32, *p* = 0.749, Cohen’s *d* = 0.04. After plotting the data, it is clear that there is an outlier, who has a time cost that is above three standard deviations from the mean. In order to ensure that the outlier was not driving the difference in search time, the data were re-analyzed without this data point. In this analysis, the average difference in search time between interruption (M = 141.23, SD = 52.92) and control trials (M = 130.04, SD = 52.04) is 11.2 s (median = 10.1 s, range = –40 to 69 s), t(25) = 1.96, *p* = 0.06, Cohen’s *d* = 0.21. This result is just outside the threshold for statistical significance. However, the effect size is approximately the same magnitude as the observed effect in Experiment 1 (Cohen’s *d* = 0.22). Therefore, the outlier does not seem to meaningfully change the outcome of the study.

#### Accuracy

Overall, 68% of the nodules were detected (see Fig. 5b). As in Experiment 1, there were no differences in the number of nodules missed between interruption (M = 2.24, SD = 0.88) and control trials (M = 2.26, SD = 0.95), t(26) = 0.15, *p* = 0.88, Cohen’s *d* = 0.02. The number of missed nodules per lung did not differ between easy (M = 2.23, SD = 0.85) and hard (M = 2.25, SD = 1.05) interruptions, t(26) = 0.16, *p* = 0.87, Cohen’s *d* = 0.03.

#### Working memory

The average working memory capacity was 2.37, SD = 1.16. Working memory capacity was not a significant predictor of time cost, r(25) = 0.14, *p* = 0.486 (see Fig. 5c). If the outlier’s data are excluded in the analysis, this relationship is even smaller, r(24) = –0.06, *p* = 0.77.

## Discussion

Across both of our studies, the primary cost of interruptions was an increase in task completion time. Despite spending more time searching the interrupted cases, there were no differences in accuracy or lung coverage across the two conditions. This time cost appears to be driven by impaired memory for previously searched regions of the lung. Using generous criteria for accurate search resumption, participants were no better than chance at faithfully resuming their search. The moments immediately following the interruption were characterized by an increase in refixation rate that returned back to baseline as the search continued. This time period likely reflects the source of the extra search time, as there was a significant correlation between time cost and refixation rate in the time period immediately following the interruption. Rather than continuing search in a new region of the image, revisiting areas of the lung that have already been examined results in wasted time. In Experiment 2, we found that the observed time cost was not modulated by the difficulty of the interruption task and had no relationship with individual differences in working memory capacity.

Given the relationship between interruptions and errors observed in other types of tasks, it is somewhat surprising that we did not find this effect (Altmann, Trafton, & Hambrick, 2014; Li, Blandford, Cairns, & Young, 2008; Westbrook et al., 2010). In applied settings, interruptions have most often been studied in sequential computer tasks, where errors are typically step omissions or repetitions (Trafton, Altmann, & Ratwani, 2011). However, very little research has been done in the realm of visual search, which has fundamentally different types of errors. One possible explanation for our results is that participants engaged in a speed/accuracy tradeoff in which errors were prevented by sacrificing time. This type of tradeoff can be experimentally induced in an interrupted data-entry task by extending the resumption lag or manipulating the time-cost penalty (Brumby, Cox, & Back, 2013). In our task, participants were instructed to be as accurate as possible and no emphasis or constraints were placed on completion time. Although search was impaired in the moments following the interruption, the same degree of lung coverage could be achieved by spending more time on the image as a whole. Unfortunately, unconstrained time is a luxury that is not available in the radiology reading room and institutions are often evaluated on their report-turnaround time. If there is a finite amount of time available for each case, the reader might terminate search prematurely and miss diagnostically relevant information. Future studies will aim to shed light on the relationship between speed and accuracy costs in interrupted medical image search.

In the second experiment, we found that the difficulty of the interruption task did not modulate the interruption cost. According to the Memory for Goals model, the disruptiveness of interruptions can be understood in terms of their effect on the activation level of goal-relevant information (Altmann & Trafton, 2002). Therefore, interruptions that allow relevant information to be rehearsed should be less costly (Monk et al., 2004). In many paradigms, the difficulty of a task is confounded with whether there is an opportunity for rehearsal. However, one study successfully disassociated these accounts by using three types of interruptions: number shadowing, a 1-back working memory task, and a 3-back working memory task (Cades et al., 2007). If task difficulty is the sole predictor of interruption cost, there should be a linear increase in time cost as task difficulty increases (shadowing < 1-back < 3-back). However, the two working memory tasks were equally more disruptive than the shadowing task. This suggests that it is the ability to rehearse task-relevant information, rather than solely task difficulty, that predicts the cost of interruptions. The goal rehearsal account may be able to explain the lack of a difficulty effect in our research. In visuospatial tasks, rehearsal seems to occur through shifts of spatial attention to memorized locations and performance is impaired by secondary tasks that require spatial attention to divert away from these locations (Awh et al., 1999; Awh, Anllo-Vento, & Hillyard, 2000; Awh, Jonides, & Reuter-Lorenz, 1998). A large body of evidence shows that mathematics and spatial attention are closely related and even our simple math problems appeared to disrupt the ability to maintain this spatial information (Dormal, Schuller, Nihoul, Pesenti, & Andres, 2014; Hartmann, Mast, & Fischer, 2015; Hegarty & Kozhevnikov, 1999).

In addition to the characteristics of the tasks, we should also consider which qualities of an individual serve to modulate the effects of interruption. Across both of our studies, there was considerable variation in time cost across individuals and a few participants were not negatively influenced by the interruptions at all. We hypothesized that working memory capacity would influence how susceptible an individual is to the effects of interruption. Successful resumption of a task requires that task-relevant information is held in memory throughout the interruption. In the case of suspended visual search, it is helpful to remember where you have already looked in the image and where you plan to look next. According to the Memory for Goals model (Altmann & Trafton, 2002), the task should be resumed more quickly if this information is kept active in memory. However, we found no evidence that visuospatial working memory capacity had any influence on the magnitude of the time cost in our task. This is surprising considering the relationship of working memory capacity to the variation in a number of other cognitive measures (Engle, Tuholski, Laughlin, & Conway, 1999). Furthermore, working memory has been linked to the magnitude of the interruption cost in other types of tasks (Drews & Musters, 2015; Foroughi, Werner, McKendrick, Cades, & Boehm-Davis, 2016). It is unclear why we did not observe this relationship in our study. One possibility is that our sample size was not large enough to detect the effect. Alternatively, it is possible our task did not sufficiently tap into memory for spatial locations. A spatial working memory task might yield a stronger relationship than the change detection task, which primarily measures memory for object features. Although performance in the change detection task is moderately correlated with measures of spatial working memory (Unsworth, Fukuda, Awh, & Vogel, 2015), this issue merits further exploration with larger sample sizes in future research.

One limitation to this study is the unrealistic nature of the interruption task. Radiologists are not likely to be interrupted with an urgent call to solve math problems. They are far more likely to be interrupted by a phone call from a colleague with a question about another patient (Ratwani et al., 2016). In order to resolve the question, the radiologist might pull up medical images from another patient, which would effectively interleave two or more visual search tasks. The interruptions in our study were limited in frequency to once per image and relatively short in duration (~40 s for easy interruptions compared to 2.4 min in the real world, according to Ratwani et al., 2016). Furthermore, real cases typically consist of several images and much larger CT scans than the images used in our study. As the number and size of the images increases, interruptions likely become more frequent and recovery might become more difficult. The effects we observed in our study were fairly small. However, these factors suggest that our interruptions may have been relatively less disruptive than the interruptions that occur in the radiology reading room. Nonetheless, further work is needed to determine how the magnitude of the interruption cost is influenced by these factors in applied settings. Another limitation of this study is that our participants were undergraduate students with no medical experience. The characterization of expert search through volumetric images is still in its infancy, but there is substantial literature demonstrating that radiologists search through 2D medical images in a qualitatively different manner than novices (for a review, see Krupinski, 2000). Radiologists also benefit from being more familiar and comfortable with interpreting medical images, which might allow task-relevant information to be more strongly encoded for later recall. Although it is unlikely that experts are immune to the effects of interruption, this research should be considered with this limitation in mind until more ecologically valid studies are completed using experts.

Faced with frequent interruptions and their repercussions, the most prudent course of action is to reduce sources of interruption in the workplace. This approach has been quite successful in aviation, where sterile cockpit rules are implemented during critical time periods. However, there is limited evidence that attempts to eliminate interruptions in healthcare (e.g. quiet zones or do not disturb signals) have had a positive impact on patient safety (Raban & Westbrook, 2014). Unlike the relatively controlled environment of a cockpit, it may be unrealistic or even misguided to completely eliminate interruptions in complex medical environments that rely on intercommunication for patient care (Durso & Drews, 2010; Rivera-Rodriguez & Karsh, 2010). In situations where interruptions cannot be eliminated, we can examine the empirical evidence for ways to mitigate the associated cost. Our study suggests that maintaining a representation of where you have looked in an image is easily disrupted by a secondary task. Even under ideal conditions, explicit memory recall for previously viewed locations is imperfect. Observers are able to reliably distinguish their own eye movements from randomly generated scan paths, but they are only able to discriminate from the eye movements of others at a level just above chance (Foulsham & Kingstone, 2013). This pattern of results suggests that recalling your own eye movements may be largely due to educated guesses based on scene semantics rather than the ability to remember where you have looked (Võ & Wolfe, 2015). Although explicit recall is poor, there is evidence for the role of mnemonic and attentional processes in guiding search toward novel locations and aiding in the resumption of a suspended task (for a review, see Shore & Klein, 2000). However, complex interruptions seem to disrupt these processes and cause search to resume in locations that have already been examined. If impaired memory is the cause of the extra search time, eye-tracking feedback or placeholders may be able to aid search resumption by offloading this information onto the environment. The Memory for Goals model predicts that priming task-relevant information through environmental cues should aid in task resumption. Consistent with this prediction, many studies have found that providing environmental cues for where in space a task was suspended lessens the time cost of an interruption (Altmann & Trafton, 2004; Trafton et al., 2005). In future research, we hope to determine whether this approach can be applied in a radiology setting.

## Conclusions

Interruptions are ubiquitous in radiology, but their effects on medical image interpretation are still largely unknown. This study is the first to quantify the cost of these interruptions using an experimental design. The interrupted CT scans were searched 8–13% longer, but there were no differences in accuracy or lung coverage. Through the use of eye-tracking, we were able to gain a better understanding of why interruptions led to an increase in task completion time. In the moments following the interruption, the observers seemed to be lost in these large, volumetric images. When interruptions are unavoidable, targeted interventions such as eye-tracking feedback or placeholders may be able to reduce the time it takes to recover from an interruption. Future work is needed to replicate these results in experts and determine how different types of interruptions influence search behavior.

## Abbreviations

CT: Computed tomography
PET: Positron emission tomography
RT: Response time
UFOV: Useful field of view
